# Breast Cancer Heterogeneity and Response to Novel Therapeutics

**DOI:** 10.3390/cancers12113271

**Published:** 2020-11-05

**Authors:** Mariona Baliu-Piqué, Atanasio Pandiella, Alberto Ocana

**Affiliations:** 1 Experimental Therapeutics Unit, Medical Oncology Department, Hospital Clínico San Carlos (HCSC), Instituto de Investigación Sanitaria San Carlos and CIBERONC, 28040 Madrid, Spain; maria.baliu@salud.madrid.org; 2 Instituto de Biología Molecular y Celular del Cáncer and CIBERONC, CSIC-IBSAL, 37007 Salamanca, Spain; atanasio@usal.es; 3 Translational Oncology Laboratory, Centro Regional de Investigaciones Biomedicas, Castilla-La Mancha University (CRIB-UCLM), 02008 Albacete, Spain

**Keywords:** breast cancer, heterogeneity, drug resistance, targeted therapies

## Abstract

**Simple Summary:**

Breast cancer is a heterogeneous disease that is driven by genetic, epigenetic and phenotypic modifications and is also affected by the microenvironment and the metabolism. In this article we review genetic and non-genetic causes of tumor heterogeneity focusing on the impact that heterogeneity has on resistance to therapy. We will provide examples of personalized medicines and their translation to the clinic.

**Abstract:**

Targeted cancer therapies against oncogenic drivers are actively being developed and tested in clinical trials. Targeting an oncogenic driver may only prove effective if the mutation is present in most tumoral cells. Therefore, highly heterogeneous tumors may be refractory to these therapies. This makes tumor heterogeneity a major challenge in cancer therapy. Although heterogeneity has traditionally been attributed to genetic diversity within cancer cell populations, it is now widely recognized that human cancers are heterogeneous in almost all distinguishable phenotypic characteristics. Understanding the genetic variability and also the non-genetic influences of tumor heterogeneity will provide novel insights into how to reverse therapeutic resistance and improve cancer therapy.

## 1. Phenotypic Features of Tumor Heterogeneity

Tumors cannot be considered as homogeneous and static entities. Tumor heterogeneity can be assumed for virtually all distinguishable phenotypic features of a tumor, that is, cellular morphology, gene expression, hormonal receptors, growth factors, cell surface markers, metabolism, motility, immunogenicity, proliferation capacity and the potential to metastasize and to promote angiogenesis [1,2,3,4]. It is recognized that tumor heterogeneity is associated with poor prognosis and survival [5,6] and is one of the leading determinants of therapeutic resistance and treatment failure [7,8].

Tumor heterogeneity comes in different flavors. First, heterogeneity may arise among the cells of one individual tumor, the so-called intratumoral heterogeneity [9,10]. Intratumoral heterogeneity may exist across different regions of the primary tumor, spatial heterogeneity and as variations of a primary tumor over time, temporal heterogeneity [11]. Second, intermetastatic heterogeneity is the variety between different metastatic lesions of the same patient [9,10] and can arise even when tumor cells in distant sites share a common ancestor, since specific factors from each metastatic site, for example, tumor microenvironment, may induce divergence after initial colonization of distant different sites [11]. Third, heterogeneity may also be present within the cells of an individual metastasis. Intrametastatic heterogeneity may or may not impact the initial response to therapy but is likely to be responsible for disease recurrence after an initial response. Such recurrences result from mutations present in a small fraction of the cells within each metastasis either prior to treatment or as a consequence of it. Usually, the larger the lesion, the more likely that such resistant cells will exist or evolve [10]. Each metastasis is established by a single cell (or small group of cells) with a set of founder genetic alterations and it acquires new mutations as it grows [9,10]. Finally, heterogeneity also exists among the tumors of different patients, which requires personalized treatments adapted to each individual [10]. Because cancer is a heterogeneous dynamic disease, individual patients, lesions and sites should be characterized over time to assure personalized treatments adapted to target the molecular drivers.

In this article, we review genetic and non-genetic causes of tumor heterogeneity focusing on the impact that heterogeneity has on resistance to therapy. We discuss in detail how breast cancer heterogeneity is driven at the genetic, epigenetic and phenotypic level, the influence of the microenvironment and the metabolism and its specific role in resistance to anti-cancer therapies.

## 2. Genomic Indications of Tumor Heterogeneity

Early histologic and biochemical studies in breast cancer showed that breast tumors are composed of different subpopulations. Breast tumors were, therefore, classified according to the expression of several clinical biomarkers, such as estrogen (ER) and progesterone (PR) receptors and the human epidermal growth factor receptor 2 (HER2) gene amplification [12,13,14]. This subclassification stands as one of the major determinants dictating current therapy of breast tumors.

Transcriptomic studies classified breast tumors into four intrinsic subtypes with distinct clinical outcomes, namely luminal A, luminal B, HER2-enriched and basal-like [15]. Alternative classifications also exist; for example, some authors subdivided triple negative breast cancers (TNBC) into six subgroups: basal-like 1, basal-like 2, immunomodulatory, mesenchymal, mesenchymal stem-like and luminal androgen receptor subtype, demonstrating that heterogeneity at a transcriptomic level is clearly evident [16]. Additional data in this breast cancer subtype, using single cell level studies with a nanogrid single-nucleus RNA-sequencing technology, showed that even most cells displayed a basal-like subtype, a significant fraction of cells were HER2+, luminal A, luminal B and normal-like, reflecting breast tumor heterogeneity even within a single breast cancer subtype [17]. Recent studies based on genome sequencing, such as the Molecular Taxonomy of Breast Cancer International Consortium (METABRIC) [18,19] and The Cancer Genome Atlas Network (TCGA) [20] refined the genomic characterization of breast cancers. Moreover, sequencing studies highlighted the genetic differences between primary tumors and metastases [21]. Genomic studies at single cell level showed profound genetic heterogeneity and extensive clonal diversity in breast cancer [22,23], as well as heterogeneity in copy number alterations of genes and regions with known biological relevance in breast cancer, such as genes associated with metastasis and therapeutic response [24].

These studies and others not reviewed here demonstrate the heterogeneity of breast tumors and emphasize the importance of molecular-based stratification of breast cancer patients in an intent to select the right therapeutic strategy.

## 3. Genetic Heterogeneity and Personalized Medicine

Driver-molecular alterations are those that directly promote tumor generation/progression. Heterogeneity of driver-gene alterations, either intratumoral, intermetastatic or intrametastatic, determine the capability of a tumor to respond to a given targeted therapeutic agent. If a single clone lacks the driver-gene mutation being targeted, the clone will likely continue to grow even when the therapy is initiated. Analyses of several genomic databases have suggested that there are around 40–60 recurrent driver-alterations in breast cancer [25]. Breast tumors may present more than ten somatic mutations. Of these mutations, only a few are present in the same genes in two different breast cancer patients. In most cases, these shared mutations correspond to driver-gene mutations [20,26,27].

Recent advances in the detection of genomic alterations and the increasing availability of genomic tests have provided relevant information about tumor heterogeneity. Such advances aid in the prediction of clinical treatment benefit and the identification of treatment resistance [28]. For example, the presence of genomic alterations in genes such as *ERBB2*, *PIK3CA*, *AKT1*, *ESR1* and *NTRK* in advanced breast cancer patients help to stratify patients for targeted therapies [28]. The European Society for Medical Oncology (ESMO) has created the ESMO Scale for Clinical Actionability of Molecular Targets (ESCAT) and classified molecular abnormalities in six levels according to the evidence of clinical benefit demonstrated by targeting the specific alteration [29]. The alteration-targeted therapy which is associated with a clinical benefit based on improved outcome in clinical trials is ranked in the 1st level. Patients harboring level 1 alterations should have access to the targeted therapy as standard of care [25]. *ERBB2* amplification, germline *BRCA* 1/2 mutations, *PIK3CA* mutations, *NTRK* translocations and microsatellite instability (MSI) were included in the 1st tier of evidence (Table 1) [25].

## 4. Heterogeneity and Resistance to Anti-Cancer Therapies

### 4.1. Heterogeneity in Target Expression

The incorporation of targeted therapies into clinical practice has improved the prognosis of certain subgroups of patients with solid tumors, however, relapse may occur due to the presence of resistance mechanisms. Resistance present in the initial tumor is known as intrinsic or primary resistance. When resistance appears after an initial response, it is named secondary resistance. Two forms of secondary resistance are acknowledged. Acquired resistance refers to secondary resistance that raises during treatment, due to acquisition of additional molecular alterations, such as mutations, activation of bypass signaling pathways and cell lineage changes [11,30]. Another type of secondary resistance, termed intrinsic resistance, appears by the outgrowth of resistant clones present pre-treatment and selected to grow by pressure on other therapy-sensitive tumoral cells [11,31,32].

Most of the targets considered as druggable are not expressed homogeneously within the tumor. For instance endocrine therapy, such as Fulvestrant or Tamoxifen, is an effective targeted therapy for ER-positive breast cancer. However, its activity depends on the fraction of ER positive cells within a tumor and this varies widely. The recommended cutoff to distinguish ER-positive patients which will receive endocrine therapy is ≥1% ER positive tumor cells [33]. ER expression is a critical predictor of response to endocrine therapy, therefore it is not surprising that lack of ER expression by some clones results in resistance to therapy [34].

Target heterogeneity is also a main issue in antibody–drug conjugate (ADC) therapy. ADCs are constituted by an antibody linked to a cytotoxic agent. Heterogeneity in the expression of the target may lead to ADC resistance due to the inability of the ADC to kill cells that do not express the target [35]. Patients with breast cancers overexpressing HER2 or with amplification of *ERBB2*, which include 20–25% patients, clinically benefit of regimens combining chemotherapy and HER2-targeted agents [36]. Trastuzumab Emtansine (T-DM1), a HER2-targeted-antibody–drug conjugate with a tubulin inhibitor payload (DM1), is an effective therapy for advanced metastatic disease as well as in the adjuvant setting. However, T-DM1 is only effective against cells expressing HER2. T-DM1 resistance appears through the selection of clones with no or limited expression of HER2 [37]. Moreover, a subgroup of HER2-overexpressing tumors may express truncated forms of HER2, which lack the region recognized by Trastuzumab, therefore impeding binding of T-DM1 [37,38]. To overcome such resistances, ADCs may be designed to exert a bystander killing effect, that is to kill cells which express the target molecule as well as surrounding cells irrespective of the expression of the target [35]. The bystander killing effect is of particular importance since it allows the delivery of the payload in areas where resistance clones outgrow due to the lack of inhibitory pressure. A different Trastuzumab-derived ADC, Trastuzumab deruxtecan (Enhertu, DS-8201) has recently been granted an accelerated FDA approval for the treatment of unresectable or metastatic HER2-positive breast cancer patients who have received at least 2 prior lines of anti-HER2-based regimens in the metastatic setting [39,40]. DS-8201 is an HER2-targeted ADC with a potent topoisomerase I inhibitor payload which presents bystander killing [41] and showed antitumor efficacy against several breast cancer models with low HER2 expression [42]. Another interesting feature of ADCs is that their bystander killing effect can be modulated by the chemical properties of the ADCs. For example, newly designed ADCs deliver the drug in response to changes in the pH [43].

Another example of target heterogeneity is the heterogeneous expression of PD-L1 in breast cancer, which can vary up to 4-fold in different areas of the same biopsy [44,45]. Patients with metastatic or locally advanced TNBC with PD-L1 expression on immune cells occupying ≥1% of tumor area have demonstrated survival benefit with combined therapy of Atezolizumab and Nab-Paclitaxel [46]. Similar results were obtained recently with Pembrolizumab, confirming that patients expressing PD-L1 obtain better outcomes [47]. Moreover, it has been shown that PD-L1 expression remains largely unaltered after neoadjuvant chemotherapy [48,49], raising the possibility that adjuvant immune checkpoint inhibitor therapy could improve survival in this patient population. In this regard, recent data from neoadjuvant therapy in combination with Pembrolizumab has shown an increase in pathological complete response, compared with combinations without the immune checkpoint inhibitor [50].

### 4.2. Clonal Selection and Resistance

Exposure to targeted therapies often gives rise to mutations or genomic modifications in metastases that justify the progression or the appearance of recurrences. The classic example of resistance to targeted therapies is the introduction as first-line treatment of the Bcr-Abl selective tyrosine kinase inhibitor (TKI) Imatinib Mesylate for chronic myeloid leukemia (CML). Although initial responses were high, therapy failed in a substantial proportion of patients and initial responses were lost within 2 years in approximately half of patients due to the development of resistances. Resistance to this TKI depends on genomic mechanisms, such as point mutations in the Bcr-Abl kinase domain, as well as on Bcr-Abl-independent mechanisms, including activation of alternative signaling pathways or insensitivity of pre-exiting clones which promotes their selection [51,52].

For breast cancer, there are also several examples of clonal selection and resistance. Single-cell DNA-sequencing of 20 TNBC before and after neoadjuvant chemotherapy showed that resistant cells are present in the tumor before the initiation of therapy and that adaptive resistance to neoadjuvant chemotherapy largely comes from the selection of pre-existent clones [53]. Similarly, DNA and RNA sequencing and live cell imaging plus single-cell RNA-sequencing of metastatic ER+ breast cancer patients showed that pre-existing minor subclones become dominant after chemotherapy, indicating selection for resistance phenotypes [54,55], similarly to what was initially described for CML, lung cancer and melanoma [56].

Approximately 30% of breast cancer patients treated with Tamoxifen become refractory within 2–5 years or develop resistance to the drug along treatment [57]. Acquired mutations in the *ESR1* gene, which encodes the ERα, alter the steroid hormone ligand-binding domain, restore the pro-oncogenic function of the ER and reduce or abrogate the therapeutic effect [58,59]. Moreover, aberrant methylation of CpG islands located in the 5′ regulatory regions of *ESR1* gene has also been associated with loss of ER expression and, hence, with acquired resistance [34].

### 4.3. Presence of Compensatory Signaling Pathways

Acquired resistance to targeted drugs may also occur via the activation of alternative signaling pathways [37,60]. One of the Trastuzumab and T-DM1 mechanisms of action is the inhibition of the PI3K signaling pathway. Therefore *PIK3CA* and *PTEN* mutations may contribute to the acquisition of resistance to these agents [37,60,61]. Moreover, EGFR, HER3, HER4, IGF-1R, MET upregulation and heterodimerization may confer resistance to anti-HER2 therapies by restoring the original downstream signaling pathways activated by HER2-overexpression and amplification [61,62,63].

### 4.4. Genomic Instability

Defects in DNA repair pathways, for example through mutations in *BRCA1*, *BRCA2* and *PALB2*, enable cancer cells to accumulate genomic alterations that contribute to their aggressive phenotype [64]. However, tumors rely on residual DNA repair capacities to survive DNA damage. Members of the poly-adenosine diphosphate–ribose polymerase (PARP) family of enzymes are central to the repair of single-strand DNA breaks. Inhibitors targeting PARP have shown promising clinical activity in tumors carrying germline mutations in either *BRCA1* or *BRCA2* [65,66,67,68]. The PARP inhibitor Olaparib is approved by the FDA for the treatment of *BRCA*-mutated breast, ovarian and pancreatic cancers. Olaparib inhibits PARP enzymes and traps PARP1 on DNA at single-strand breaks, leading to replication-induced DNA damage. Such damage needs to be repaired by homologous recombination, which makes cancer cells defective in DNA repair pathways highly sensitive to Olaparib [69,70].

As is the case for many other treatments, tumors frequently acquire resistance to PARP inhibitors [71]. Four distinct categories of resistance mechanisms have been described: (i) restoration of the homologous recombination mechanisms; (ii) decreased availability of PARP, for example, via point mutations in PARP1 [72]; (iii) increased drug efflux; and (iv) restoration of replication fork stability [73]. The restoration of the homologous recombination mechanisms mainly arises from reverting mutations in *BRCA1* or *BRCA2* genes that restore the open reading frame of the genes. Such mutations also cause clinical resistance to platinum-based chemotherapy [74,75]. The fact that polyclonality of multiple reverting mutations emerges within one patient illustrates the profound selection pressure exerted on these tumors to restore BRCA1/2 protein activity and acquire resistance [73]. 

### 4.5. Epigenetic Modifications

Besides genetic heterogeneity, human tumors usually contain epigenetic changes. Epigenetic modulation plays a critical role in regulating where and when genes are expressed during tumor development [76]. Gene promoter methylation, general hypomethylation and histone methylation and deacetylation are common in cancer. Such epigenetic alterations exert a selective effect on clones presenting a specific epigenetic event, such as the inactivation of tumor suppressor genes by promoter methylation [77]. Moreover, epigenetic heterogeneity also allows for reversible transitions from drug-sensitivity to drug-resistance [30].

In basal-like breast cancer, tumor cell populations, which persist following treatment with the MEK inhibitor, Trametinib, and the PI3K/mTOR inhibitor, BEZ235, showed increased activity of BRD4, KDM5B and EZH2 [78]. In addition, the genes involved in SWI/SNF chromatin remodeling complex, including ARID1A, ARID1B and ARID2, have been found to be mutated in metastatic recurrences of treatment-resistant breast cancer patients [79]. These observations provide evidence that epigenetic changes can generate drug-resistant states that allow for the survival of small subpopulations within otherwise treatment-sensitive cancer cells. Therefore, drugs targeting epigenetic enzymes may decrease intratumoral cellular heterogeneity and reverse treatment resistance, when combined with chemotherapy or targeted therapies.

A beautiful example of the role of epigenetic modifications in drug resistance is the modulation of epigenetics to generate drug sensitization. In animal models of breast and ovarian cancer, the inhibition of Bromodomain and Extra-Terminal motif (BET) proteins impairs the transcription of *BRCA1* and *RAD51*, two genes essential for homologous recombination and increases the tumor sensitivity to Olaparib [80]. Other example from our laboratory is the synergy observed when treating PLK1-resistant cells with the BET inhibitor JQ1 [81].

### 4.6. Tumor Microenvironment and Immune System

The tumor microenvironment (TME)—the space surrounding the tumor composed of immune cells, stroma and vasculature—also plays a role in resistance to therapy [82]. To understand tumor progression and the appearance of resistances to targeted therapies it is important to recognize the multiple components of the TME and the interactions between the tumor and its surrounding. The TME may create different selective pressures in distinct tumor areas and this may give rise to intratumor heterogeneity and favor the outgrowth of specific clones [83,84]. Hypoxia, inflammation and the fibrotic state of the tissue can directly and indirectly influence tumor heterogeneity [30,83]. Hypoxia is one of the most well-known examples, hypoxic conditions may trigger a set of adaptive transcriptional responses, including cell metabolism, invasion, survival, angiogenesis, differentiation and self-renewal, that seem to be involved in tumor progression and in the expression of drug-resistance genes [85,86,87,88]. Furthermore, hypoxia and inflammatory cytokines released by stromal or immune cells can induce epigenetic modifications that subsequently alter gene expression [8].

Fibroblasts constitute one of the most abundant cell types in the stroma. It is recognized that fibroblasts can apply suppressive functions on tumor cells. However, during tumor progression, fibroblasts loss their suppressive effect and allow tumor growth [89]. Cancer-associated fibroblasts (CAFs) are a heterogeneous cellular population that can alter the tumor response to therapy and the immune response. Four subsets of CAFs have been characterized in breast cancer [90]. The CAF-S1 subset, which is enriched in TNBC, is known to support an immunosuppressive environment [90].

The presence of tumor infiltrating lymphocytes (TILs) is associated with favorable outcome in breast cancer [91,92,93]. However, there is heterogeneity in subset composition, functional status and spatial location of immune cells within the tumor [94,95,96]. ‘Cold’ tumors have few immune cells, largely macrophages; mixed tumors harbor immune cells and tumor cells mixed together. In compartmentalized tumors, the immune and tumor cells are spatially segregated [95]. Around 70% of breast tumors are infiltrated with TILs, with a median TIL count of around 10% [48,49]. In breast tumors, PD-L1 is mainly expressed in stromal cells, including TILs, macrophages and morphologically fibroblast-like cells, while cancer cells express PD-L1 only in half the cases [48,97]. This pattern of expression observed in breast cancer implies PD1/PD-L1 signaling between tumor cells and immune cells, as well as between various types of immune cells, and is important in the action of PD1/PD-L1 targeting antibodies [98]. Recently, several transcriptomic signatures associated with the presence of immune infiltrates in breast tumors have been described [99,100]. Such signatures identify ‘hot’ tumors what could potentially predict response to immunotherapies.

The TME also differs between primary tumor and metastases, which influences the phenotype of tumor cells at distal sites [101]. Metastatic lesions in breast cancers have been shown to be less immunologically active [102]. Metastasis may escape from immune surveillance by down regulating chemotactic and immune activating cytokines and their receptors, decreasing antigen presentation and upregulating immunosuppressive mechanisms [102]. The presence of TILs and the expression of PD-L1 is substantially lower in metastases compared with primary tumors [102], a situation which could impair the response of metastatic lesions to immune checkpoint inhibition with anti PD1/PD-L1 antibodies.

Moreover, it is now well recognized that tumor-normal cell fusions, the so called tumor-normal hybrids, are potent inducers of genomic instability and heterogeneity and that they contribute to resistance to therapy [103,104]. Cell fusion has been observed between breast tumor cells themselves [105], between tumor cells and normal breast epithelium [106], endothelial [107], stromal cells, stem cells [108] and macrophages [109,110].

Heterogeneity in the TME, the immune system and the presence of tumor-normal cell hybrids may directly and indirectly affect response to therapy and resistance to drugs. Immunosuppressive cancer microenvironments and immune desert tumors are the major impediments to checkpoint inhibitors. Some immunotherapy-resistant tumors have a low mutational burden, which translates to low antigen presentation and low tumor immunogenicity [111,112]. Other mechanisms of resistance to immune checkpoint blockade include the loss of β2-microglobulin, which impairs antigen presentation, and JAK1 or JAK2 mutations which make tumor cells insensitive to interferon gamma [113]. Recent studies have identified genomic correlates of response to immune checkpoint blockade which may help identify immunotherapy responsive tumors [114,115,116].

### 4.7. Metabolism

Besides its heterogeneous composition, the TME may also be a metabolic barrier to effector T cells and immunotherapy [117]. The TME can be metabolically hostile (hypoxic, depleted of nutrients and with accumulation of waste products) for T cells and lead to metabolic dysfunction. T cells require an adequate nutrient uptake to mount a proper immune response and the lack of sufficient nutrients or the failure to activate the right metabolic pathways may prevent effector T cell activation [117]. Effective antitumoral responses by T cells require optimal T cell fitness and circumstances to avoid exhaustion. PD1 signaling on T cells may induce the metabolic switch from glycolysis to fatty acid oxidation which impairs the effector function of T cells [118,119,120,121]. These observations suggest that anti-PD1 treatment may be able to restore T cell glycolysis and, subsequently, the effector function of T cells.

Moreover, tumor cells also secrete byproducts that may be harmful for T cells. For example, indoleamine 2,3-dioxygenase (IDO) catalyzes tryptophan, an essential amino acid for immune responses and produces kynurenine, which induces the generation of immunosuppressive regulatory T cells [122,123]. Moreover, high lactate concentrations have been shown to suppress the effector functions of CD8^+^ T cells [124].

In patients with advanced melanoma and renal cell carcinoma treated with Nivolumab, an anti-PD1 antibody, it has been shown that the serum kynurenine/tryptophan ratio increases upon treatment as an adaptive resistance mechanism associated with worse overall survival [125]. Increased kynurenine/tryptophan ratio inhibits T cell proliferation [126,127,128], and, therefore, may promote tumor immune resistance to PD1 blockade. These observations highlight the importance of metabolism in resistance to targeted therapies and advocates for metabolic monitoring of patients during immunotherapy.

## 5. Beyond Tumor Heterogeneity

Despite tumor heterogeneity, the identification of driver vulnerabilities and the development of novel targeted therapies has a meaningful clinical impact. Tumor-agnostic drugs are treatments that target a specific genetic characteristic irrespective of tumor histology (Table 2). Regardless the histologic origin, tumors presenting mismatch repair deficiency (MMRd) which exhibit MSI are highly sensitive to immune checkpoint blockade, with an overall response rate (ORR) of 36–46% across 15 different histologies and with 78% of responses after 6 months [129,130,131]. Therefore, Pembrolizumab has been the first FDA approved tumor-agnostic drug for the treatment of MSI-positive tumors [131,132,133]. Both the genomics and the TME of MSI-positive tumors contribute to the remarkable response rates: (i) MSI-positive tumors generate a great amount of neo-epitopes [129,130,131,134], which tend to be subclonal since MMRd-induced mutations are predominantly subclonal and derive in highly heterogeneous tumors [135]; (ii) MSI-positive cancers are highly infiltrated with CD8^+^ T cells [136]; and (iii) MSI-positive cancers express high levels of multiple immune checkpoint molecules, including PD1 and PD-L1 [137].

Larotrectinib, a tropomyosin receptor kinase (TRK) inhibitor, was the second agnostic drug approved by the FDA [133]. In patients presenting neurotrophic receptor tyrosine kinase (NTRK) fusions, Larotrectinib has shown an ORR of 80% across 17 tumor types, with 16% of patients having a complete response [138]. Larotrectinib regulatory approval was followed by the approval of Entrectinib, another TRK inhibitor [133]. Despite the remarkable ORR achieved by Larotrectinib treatment, resistances have already been reported, including solvent front mutations, gatekeeper mutations and mutations in the xDFG motif [139]. These mutations modify the binding site of Larotrectinib, decreasing its inhibitory potency [139]. Preclinical and *in silico* modelling were able to predict which mutations of the TRK kinase domain would drive resistance to Larotrectinib and enabled the development of novel TRK inhibitors that targeted the kinase-domain of TRK mutants [140].

Future work in tumor-agnostic drug development should look for therapeutic agents that target true driver mutations and take in consideration coexisting or newly acquired mutations that can drive resistance. Approaches to predict mutations that drive resistances, such as preclinical and *in silico* modelling, will be of clinical relevance and will likely be introduced to predict resistances to new drugs.

## 6. Conclusions and Perspectives

There is a wide range of sources of biological heterogeneity including tumor immune profiles and TME, tumor mutational burden and development of tumoral cell polyclonality along therapy. Common patterns of resistance to driver-gene targeted therapies and the presence of low-frequency drivers must be anticipated and intercepted in order to achieve a durable clinical response. We believe that in the near future cancer patients will be treated with a rationally designed combination therapy of targeted agents, rather than with a single drug. These therapies may combine several drugs aimed at targeting different oncogenic drivers present in distinct tumoral cell populations. Moreover, these therapies may be combined with others, such as those that boost immune responses against tumors. Although examples of this approach are the ongoing studies combining immunotherapies with targeted agents in selected populations, much work is still needed to achieve a personalized treatment for each patient.

## Figures and Tables

**Table 1:** List of alterations ranked as level 1 by Condorelli et al. [25].

Gene	Type of Alteration	Drug
*ERBB2*	Amplification (DNA copy number ≥6; size ≤10 Mb)	Anti-HER2 monoclonal antibody (Trastuzumab)
*BRCA 1/2* mutations	Germline mutations: Truncated mutations (InDel, splice-site, non- sense (except BRCA2 K3326X)) and rare known inactivating missense mutations	PARP inhibitor (Olaparib)
*PIK3CA*	Major hot-spot activating missense mutations	Alpha-specific PI3K inhibitor (Alpelisib)
*NTRK*	Translocations	Pan-Trk inhibitor (Larotrectinib)
-	Microsatellite instability (MSI)	Anti-PD1 antibody (Pembrolizumab)

**Table 2:** Molecular alterations with agnostic indications approved or in development [133,138].

Gene	Type of Alteration	Drug	Development Phase
	Microsatellite instability–High (MSI-H)	Pembrolizumab	FDA approved
*NTRK*	Gene fusions	Larotrectinib, Entrectinib	FDA approved
*RET*	Gene fusions/mutations	Selpercatinib Pralsetinib RXDX-105 TPX-0046	Phase I/II Phase I/II Phase I/Ib Phase I/II
*NRG1*	Gene fusions	Zenocutuzumab Tarloxotinib	Phase I/II basket
*FGFR* (fibroblast growth factor receptor)	Gene fusions/mutations	Debio 1347 TAS-120	Phase II basket
*KRAS* ^G12C^	Mutations	AMG 510 MRTX849	Phase I
*TRK*, *ROS1*, *ALK*	Mutations	Repotrectinib	Phase I/II
*BRAF*	Mutations	PLX8394	Phase I/II
